# Supplementary material for: From sequence to enzyme mechanism using multi-label machine learning
Source: BMC Bioinformatics. 2014 May 19;15:150. doi: 10.1186/1471-2105-15-150 (PMC4229970; doi:10.1186/1471-2105-15-150)
Supplement: Additional file 2 — Java code of ml2db. Additional file ml2db_code.tar.gz contains the Java source code to run the multi-label machine learning experiments and save the results to database. The code’s Javadoc is included. [file 1471-2105-15-150-S2.zip › additional file 2/ml2db/ml2db/doc/mulan/classifier/meta/thresholding/class-use/Meta.html]

Uses of Class mulan.classifier.meta.thresholding.Meta


JavaScript is disabled on your browser.


- Overview
- Package
- Class
- Use
- Tree
- Deprecated
- Index
- Help

- Prev
- Next

- Frames
- No Frames

- All Classes

## Uses of Class mulan.classifier.meta.thresholding.Meta

- Packages that use Meta

  | Package | Description |
  | --- | --- |
  |  |  |
  | --- | --- |
  | mulan.classifier.meta.thresholding |  |
- - ### Uses of Meta in mulan.classifier.meta.thresholding

    Subclasses of Meta in mulan.classifier.meta.thresholding

    | Modifier and Type | Class and Description |
    | --- | --- |
    |  |  |
    | --- | --- |
    | `class` | `MetaLabeler` Class implementing the MetaLabeler algorithm. |
    | `class` | `ThresholdPrediction` Class that learns to predict a different threshold per exampleFor more information, see |

- Overview
- Package
- Class
- Use
- Tree
- Deprecated
- Index
- Help

- Prev
- Next

- Frames
- No Frames

- All Classes
